# Molecular Changes in Prepubertal Left Ventricular Development Under Experimental Volume Overload

**DOI:** 10.3389/fcvm.2022.850248

**Published:** 2022-04-12

**Authors:** Yuqing Hu, Debao Li, Chunxia Zhou, Yingying Xiao, Sijuan Sun, Chuan Jiang, Lijun Chen, Jinfen Liu, Hao Zhang, Fen Li, Haifa Hong, Lincai Ye

**Affiliations:** ^1^Department of Cardiology, Shanghai Children's Medical Center, School of Medicine, Shanghai Jiao Tong University, Shanghai, China; ^2^Department of Thoracic and Cardiovascular Surgery, Shanghai Children's Medical Center, School of Medicine, Shanghai Jiao Tong University, Shanghai, China; ^3^Department of Pediatric Intensive Care Unit, Shanghai Children's Medical Center, School of Medicine, Shanghai Jiao Tong University, Shanghai, China; ^4^Shanghai Institute for Pediatric Congenital Heart Disease, Shanghai Children's Medical Center, School of Medicine, Shanghai Jiao Tong University, Shanghai, China; ^5^Institute of Pediatric Translational Medicine, Shanghai Children's Medical Center, School of Medicine, Shanghai Jiao Tong University, Shanghai, China

**Keywords:** aortic regurgitation, RNA-seq, left ventricle, volume overload, sarcomere, maturation

## Abstract

**Background:**

Left ventricular (LV) volume overload (VO), commonly found in patients with chronic aortic regurgitation (AR), leads to a series of left ventricular (LV) pathological responses and eventually irreversible LV dysfunction. Recently, questions about the applicability of the guideline for the optimal timing of valvular surgery to correct chronic AR have been raised in regard to both adult and pediatric patients. Understanding how VO regulates postnatal LV development may shed light on the best timing of surgical or drug intervention.

**Methods and Results:**

Prepubertal LV VO was induced by aortocaval fistula (ACF) on postnatal day 7 (P7) in mice. LV free walls were analyzed on P14 and P21. RNA-sequencing analysis demonstrated that normal (P21_Sham vs.P14_Sham) and VO-influenced (P21_VO vs. P14_VO) LV development shared common terms of Gene Ontology (GO) and Kyoto Encyclopedia of Genes and Genomes (KEGG) in the downregulation of cell cycle activities and the upregulation of metabolic and sarcomere maturation. The enriched GO terms associated with cardiac condition were only observed in normal LV development, while the enriched GO terms associated with immune responses were only observed in VO-influenced LV development. These results were further validated by the examination of the markers of cell cycle, maturation, and immune responses. When normal and VO-influenced LVs of P21 were compared, they were different in terms of immune responses, angiogenesis, percentage of Ki67-positive cardiomyocytes, mitochondria number, T-tubule regularity, and sarcomere regularity and length.

**Conclusions:**

A prepubertal LV VO mouse model was first established. VO has an important influence on LV maturation and development, especially in cardiac conduction, suggesting the requirement of an early correction of AR in pediatric patients. The underlying mechanism may be associated with the activation of immune responses.

## Graphical Abstract

A prepubertal LV VO mouse model was first established. VO has an important influence on LV maturation and development, especially in cardiac conduction, suggesting the requirement of an early correction of AR in pediatric patients. 
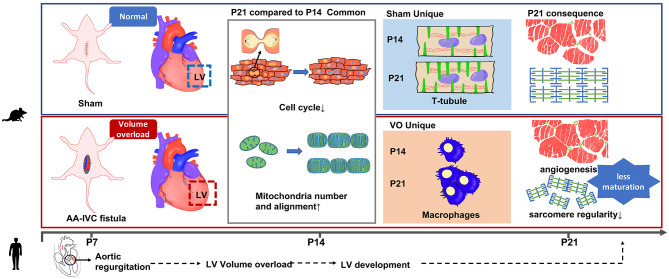

## Clinical Perspective

**Novelty:** To our knowledge, this study is the first to induce a prepubertal left ventricular volume overload mouse model and to reveal the molecular changes in prepubertal left ventricular development under volume overload.

**Clinical implications:** A platform for the study of left ventricular volume overload conditions, such as aortic or mitral valve regurgitation, is introduced. Volume overload has an important effect on left ventricular maturation and development, and this highlights the necessity of early correction of aortic regurgitation or the need for advanced drugs to support left ventricular maturation under the influence of volume overload.

## Introduction

Aortic regurgitation (AR), which produces left ventricular (LV) volume overload (VO), is documented in up to 15% of patients and ranks third in prevalence among valvular heart diseases (1–4). Children with mild or moderate AR may remain asymptomatic with normal LV systolic function for many years. However, a series of pathological responses occur in AR, and by the time patients become symptomatic, many have already developed irreversible myocardial dysfunction (5–7). In the current guidelines for the management of patients with AR, surgical intervention is recommended at the onset of symptoms or in the presence of LV systolic impairment (8–10). Thus, the applicability of this guideline in pediatric patients is now under questioning (11, 12). In addition, how VO affects pediatric LV remodeling is largely unknown (13, 14). Understanding the molecular mechanisms regulating pediatric LV development under the influence of VO may offer insights for the optimal timing of surgical intervention for AR.

Current studies have demonstrated that from postnatal day 1 (P1) to P7, rodent cardiomyocytes (CMs) are immature, with a strong proliferative potential, using glycolysis as their primary energy source (15–19). At P7, rodent CMs begin the maturation process. At P21, the CMs are fully mature, with oxidative phosphorylation as their primary energy source (15–19). From P7 to P21, the rodent heart undergoes metabolic and cardiac muscle maturation; therefore, there is an increase in sarcomere length, mitochondria (Mito) number, and T-tubule regularity (15–19). Previously, we first created a prepubertal VO model by aortocaval fistula (ACF) at P7, and using the model, we demonstrated that postnatal right ventricular (RV) maturation and development were partly altered by VO, and that the underlying mechanisms were associated with the replacement of the peroxisome proliferator–activated receptor (PPAR) signaling pathway by the cell cycle pathway (13, 18, 20). However, owing to the huge differences in embryonic origin, anatomical structure, and physiologic function between the LV and RV (21–23), the RV results cannot be applied directly to the LV, and whether VO affects LV maturation is still unexplored.

In adult animals, ACF not only induces RV VO but also produces LV VO (24, 25). In past studies, we demonstrated that ACF induces RV VO at neonatal and prepubertal animals (13, 18, 20, 26), and VO induces different responses among neonatal, prepubertal, and adult RVs (13, 26, 27), highlighting the importance of developmental stage-specific analysis of the effect of VO on RV remodeling. How ACF induces prepubertal LV VO is unknown. In the current study, we investigated whether ACF was able to induce a prepubertal LV VO, then demonstrated that the common and unique developmental processes between normal and VO-influenced LVs. Finally, the normal and VO-influenced LVs at P21 were compared to understand whether and how LV maturation is affected by VO.

## Materials and Methods

All of the RNA sequencing (RNA-seq) data have been deposited in the Gene Expression Omnibus (GEO) database (https://www.ncbi.nlm.nih.gov/geo) under the accession number GSE186968.

Information on reagents and antibodies is provided in Supplemental Table S1.

Data generated in this study are available from the corresponding author upon reasonable request.

All of the procedures conformed to the principles outlined in the Declaration of Helsinki and were approved by the Animal Welfare and Human Studies Committee at Shanghai Children's Medical Center (Institutional Review Board Approval Number: SCMCIRB-Y2020094).

### Animal Experiments

Pregnant C57/BL6 mice were purchased from Xipu'er-bikai Experimental Animal Co., Ltd. (Shanghai, China). At P7, the prepubertal mice (both male and female) were randomized into two groups—an experimental group (VO group) and a control group (Sham group)—that underwent the same procedure except for the puncture step. Male and female prepubescent mice were equally used throughout the study. The fistula surgery protocol was performed according to our previous publications (13, 18, 20, 26). Briefly, the abdominal aorta (AA) and inferior vena cava (IVC) of the prepubertal mice were exposed by a midline laparotomy under general anesthesia (4% isoflurane). A fistula between the AA and IVC was created by a puncture with a 0.07-mm diameter needle (surgical video is provided on the website: https://www.ahajournals.org/doi/suppl/10.1161/JAHA.121.020854). After the puncture, a 2-min hemostatic compression with the surrounding connective tissue was executed, and the abdominal wall was closed with pain relief (local lidocaine treatment).

### Abdominal Ultrasound and Echocardiography

At P14, the ACF and aortic valve (AoV) flow of mice were analyzed with a Vevo 2100 imaging system (Visual Sonics, Toronto, Ontario, Canada) under general anesthesia with isoflurane (isoflurane/oxygen: 1.5–2.0% maintenance). For confirmation of an ACF, the waveform in the fistula was recorded using pulse-wave mode. To confirm the VO, the velocity-time integral (VTI) of the AoV (AoV-VTI) blood flow, the AoV-velocity, LV end-diastolic volume (LVEDV), LV end-systolic volume (LVESV), and ejection fraction (EF) were calculated from the mean of three consecutive measurements using two-dimensional and pulse Doppler echocardiography.

### Histology

Hematoxylin and eosin (H&E) staining was performed with a kit according to the manufacturer's instructions (C0105M; Beyotime Biotech, Shanghai, China).

### Total RNA Preparation

Total RNA was extracted from the LV free wall and purified using a PureLink RNA micro scale kit (Catalog No. 12183016; Life Technologies, Carlsbad, CA, USA). Reverse-transcription polymerase chain reaction (PCR) was performed using the PrimeScript reagent kit (Takara Bio, Kusatsu, Japan).

### Library Preparation

Sequencing libraries were generated using the NEBNext® Ultra™ RNA library prep kit for Illumina® (New England Biolabs, Ipswich, MA, USA) following the manufacturer's recommendations. Briefly, messenger RNA was purified from total RNA using poly-T oligo-attached magnetic beads. First-strand complementary DNA (cDNA) was synthesized using random hexamer primers and M-MuLV reverse transcriptase (RNase H minus). Second-strand cDNA synthesis was performed using DNA polymerase I and RNase H. In order to select cDNA fragments of preferentially 250–300 bp in length, the library fragments were purified with the AMPure XP system (Beckman Coulter, Beverly, MA, USA). Then, 3 μl of USER Enzyme (New England Biolabs, Ipswich, MA, USA) was used with size-selected, adaptor-ligated cDNA at 37°C for 15 min, followed by 5 min at 95°C. Then, PCR was performed with Phusion high-fidelity DNA polymerase, universal PCR primers, and an index (X) primer. Finally, PCR products were purified (AMPure XP system, Beckman Coulter, Indianapolis, IN, USA), and library quality was assessed on an Bioanalyzer 2100 system (Agilent Technologies, Santa Clara, CA, USA).

### Clustering and Sequencing

The clustering of the index-coded samples was performed on a cBot cluster generation system using a TruSeq PE cluster kit v3-cBot-HS (Illumina, San Diego, CA, USA) according to the manufacturer's instructions and then sequenced on an Illumina NovaSeq platform.

### Quality Control and Read Mapping

Raw data (raw reads) in fastq format were first processed through in-house Perl scripts to generate clean data (clean reads), which were used for the downstream analyses. The paired-end clean reads were aligned to the reference genome using Hisat2 v2.0.5. FeatureCounts v1.5.0-p3 was used to count the number of reads mapped to each gene. Then, fragments per kilobase of transcript sequence per million (FPKM) of each gene were calculated based on the length of the gene and the read counts mapped to each gene.

### Differential Expression and Enrichment Analysis

Differential expression analysis was performed using the DESeq2 R package (version 1.16.1).

Gene Ontology (GO) and Kyoto Encyclopedia of Genes and Genomes (KEGG) pathway enrichment analysis was implemented by the clusterProfiler R package, which was used to correct the gene length bias. GO and KEGG terms, with corrected *P* values of less than 0.05 were considered to be significantly enriched.

### Immunofluorescence

Slides were fixed with 4% paraformaldehyde for 10 min, permeated with 0.5% Triton X-100 for 15 min, blocked with 10% donkey serum for 30 min, stained with primary antibodies (Ki67/CD31, 1:200 dilution; Abcam, Cambridge, UK) overnight at 4°C, and incubated with secondary antibodies and 4',6-diamidino-2-phenylindole (DAPI) for 30 min. The immunofluorescence images of the slides were analyzed using ImageJ software (National Institutes of Health, Bethesda, MD, USA).

### Transmission Electron Microscopy

Mito morphology and sarcomere alignment were determined by transmission electron microscopy. The LVs were removed from the chest when the mice were anesthetized with 1.5% isoflurane and cut into 1-mm^3^ pieces, and then fixed with fresh and cold 2.5% glutaraldehyde solution overnight at 4°C. The fixed samples were dehydrated, embedded in paraffin, and sectioned into 70-nm slices. Finally, the slices were scanned with JEM-1230 (80 KV).

### Flow Cytometry Analysis

To evaluate the immune cells in LVs, the LVs were minced into small fragments and dissociated with 1:1 type II collagenase (1,000 U/mL in PBS; Worthington, Lakewood, NJ, USA) and dispase (11 U/mL in PBS; Gibco Laboratories, Gaithersburg, MD, USA) at 37°C for 30 min. The dissociated cardiac cells were removed from the contaminating erythrocytes by incubation with red blood cell lysis buffer (eBiosciences, Waltham, MA, USA) for 5 min and then subsequently stained with fluorochrome-conjugated antibodies against CD4 (eBiosciences) at a dilution of 1:100 at 4°C for 30 min. Propidium iodide (PI; Becton, Dickinson, and Co., Franklin Lakes, NJ, USA)-positive dead cells were excluded for live cell analysis, and FACS data were then analyzed with the FlowJo software (FlowJo LLC, Ashland, OR).

### T-Tubule Imaging and Analysis

*In situ* T-tubule imaging and AutoTT analysis of T-tubule patterns were performed as described previously (28). Intact mice hearts were Langendorff-perfused with Tyrode's solution containing 2.5 μM of FM 4-64 (Invitrogen™, Paisley, UK) for 20 min. The hearts were placed in the perfusion chamber attached on the stage of a confocal microscope and perfused with indicator-free/Ca^2+^-free solution. The membrane structure of epicardial myocytes was analyzed *in situ* with confocal microscope with a 63× oil immersion lens. AutoTT preprocessed confocal images, and then extracted and analyzed T-tubule system morphological features.

### Sarcomere Imaging and Analysis

CMs were isolated with a Langendorff perfusion system as described previously (29, 30). After perfusion, only the LV free wall was removed and CMs from the LV were used for sarcomere imaging. Isolated CMs were fixed with 4% paraformaldehyde for 10 min, permeated with 0.5% Triton X-100 for 15 min, blocked with 10% donkey serum for 30 min, stained with sarcomeric α-actinin (SAA, 1:200 dilution; Abcam) overnight at 4°C. The images were acquired through confocal scanning using a 60× objective and analyzed by AutoTT as described previously (28). AutoTT preprocessed confocal images by background subtraction and local noise removal; extracted sarcomere morphological features; and completed the morphological feature analysis, including sarcomere length and regularity analysis.

### Statistical Analysis

Continuous data were expressed as mean ± standard deviation values. Differences were tested using the Student's *t*-test if the data were normally distributed; otherwise, they were tested with the rank-sum test. *P*-values of <0.05 were considered to be statistically significant. Statistical analyses were performed using the SAS software version 9.2 (SAS Institute Inc., Cary, NC, USA).

## Results

### Creation of ACF Mice

**Figure 1:**
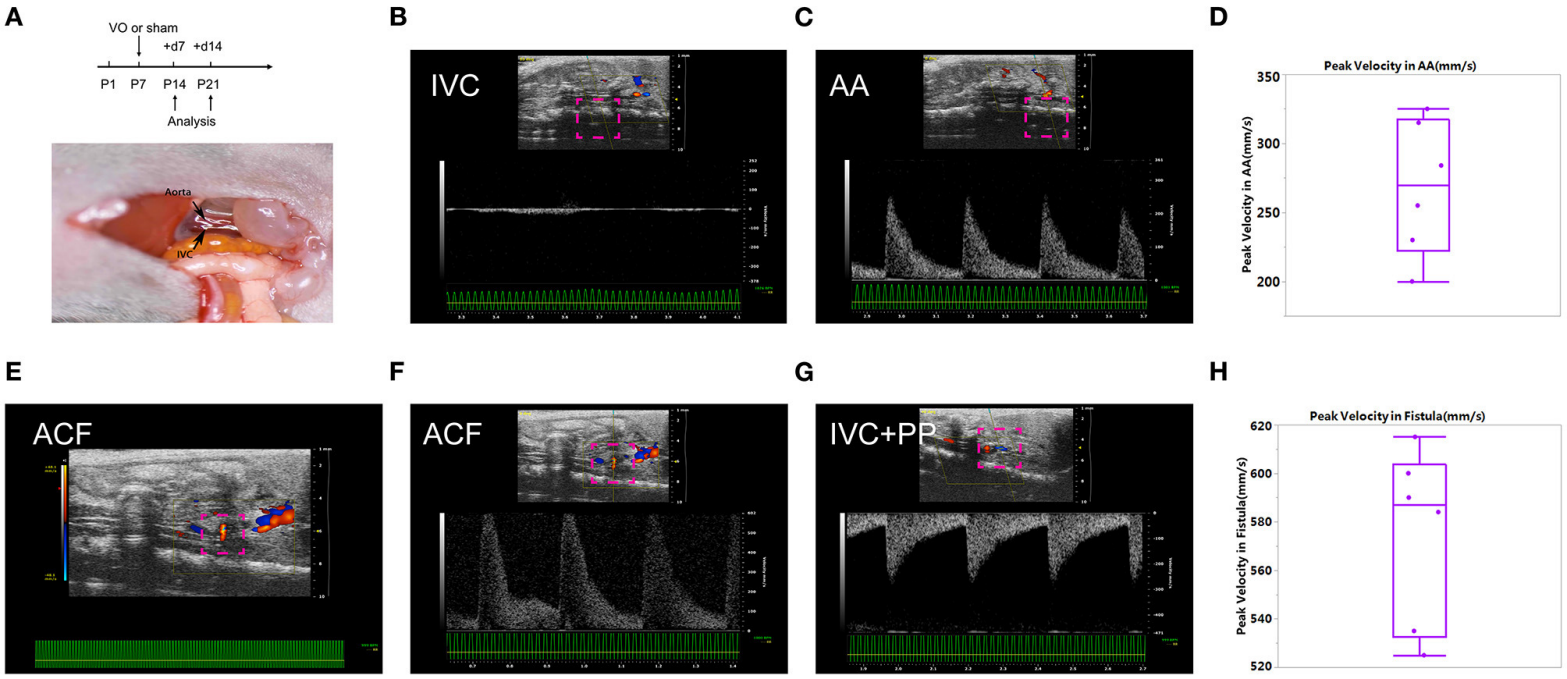
Establishment of the aortocaval fistula (ACF). **(A)** Top panel: experiment protocol; bottom penal: schematic diagram of the ACF model. **(B)** No pulsatile blood flow appears in the inferior vena cava (IVC). **(C)** Pulsatile blood flow is shown in the abdominal aorta (AA), with a peak blood flow velocity of 250 mm/s. **(D)** Quantification of peak velocity in the AA. **(E)** A representative image of blood flow through the fistula. **(F)** The representative image of pulsating blood flow at the fistula, with a peak blood flow velocity of 602 mm/s, which is higher than that of the AA. **(G)** Pulsatile blood flow appeared in the IVC after fistula establishment, with a peak blood flow velocity of 280 mm/s. **(H)** Quantification of peak velocity in the fistula. *n* = 6 mice.

As shown in Figure 1A, we conducted ACF and sham procedures on P7, and performed analyses at P14 and P21. Under normal conditions, at P14, there was no pulsatile blood flow at IVC, while with a pulsatile blood flow at the AA, the peak flow velocity up to 300 mm/s (Figures 1B,C). The average of peak velocity in the AA was 268.2 ± 48.9 mm/s (Figure 1D). At the puncture point, there was a pulsatile blood flow (Figure 1E), with a peak flow velocity up to 602 mm/s (Figure 1F). After punctuation, a pulsatile blood flow appeared in IVC, and the peak flow velocity reached up to 280 mm/s (Figure 1G). The average of peak velocity in the fistula was 574.8 ± 36.4 mm/s (Figure 1H). These results suggested that ACF mice were successfully created, consistent with our previous publications (13, 18, 20).

### Verification of LV VO in ACF Mice

**Figure 2:**
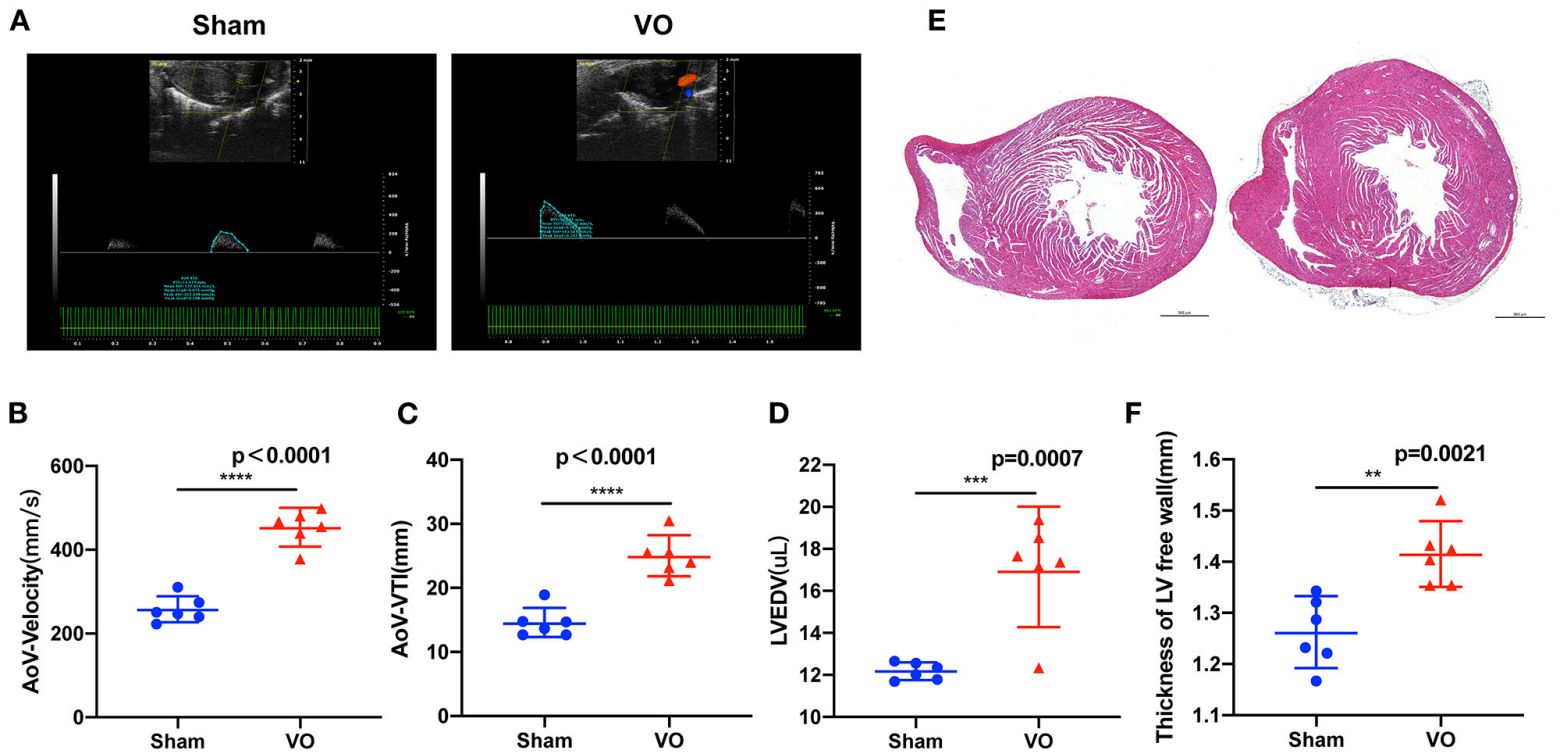
Left ventricular (LV) volume overload (VO) increased in the ACF model. **(A)** The representative echo image showed that, 2 weeks after the creation of fistula, the aortic valve (AoV) velocity and the velocity–time integral (VTI) in the VO group were increased. **(B)** Quantification of AoV–velocity in the Sham and VO groups, *n* = 6, Student's *t*-test. **(C)** Quantification of AoV–VTI in the Sham and VO groups, *n* = 6, Student's *t*-test. **(D)** Quantification of LV end-diastolic volume (LVEDV) in the Sham and VO groups, *n* = 6, Student's *t*-test. **(E)** Hematoxylin and eosin staining showed that, two-weeks after the creation of the fistula, the free wall of the LV was thickened in the VO group. **(F)** Quantification of the thickness of the free wall of the LV, *n* = 6, Student's *t*-test. **p < 0.01, ***p < 0.001, ****p < 0.0001.

To verify there was LV VO in the ACF mice, we performed echocardiography on the ACF mice at P14. The results showed that the AoV-velocities in the Sham and VO groups were 257.4 ± 30.81 mm/s and 453.3.4 ± 42.22 mm/s, respectively (*p* < 0.0001, *n* = 6), and the AoV-VTIs in the Sham and VO groups were 14.56 ± 2.325 mm and 24.98 ± 3.156 mm, respectively (*p* < 0.0001, *n* = 6) (Figures 2A–C). The LVEDVs in the Sham and VO groups were 12.18 ± 0.4051 μl and 17.08 ± 2.464 μl, respectively (*p* = 0.0007, *n* = 6) (Figure 2D). The LVESVs in the Sham and VO groups were 2.504 ± 0.7104 μl and 5.968 ± 2.342 μl, respectively (*p* = 0.006, *n* = 6) (Supplementary Figure 1A). There was no significant difference between the Sham and VO groups (Supplementary Figure 1B). To confirm the results, hematoxylin and eosin staining was performed, revealing an increased thickness of LV free wall in the VO group (Figures 2E,F). No difference between sexes was observed in any cardiac phenotype. These results confirmed that the LV VO was successfully established in ACF mice, consistent with previous publications (24, 25).

### Postnatal LV Developmental Track Is Changed by VO

**Figure 3:**
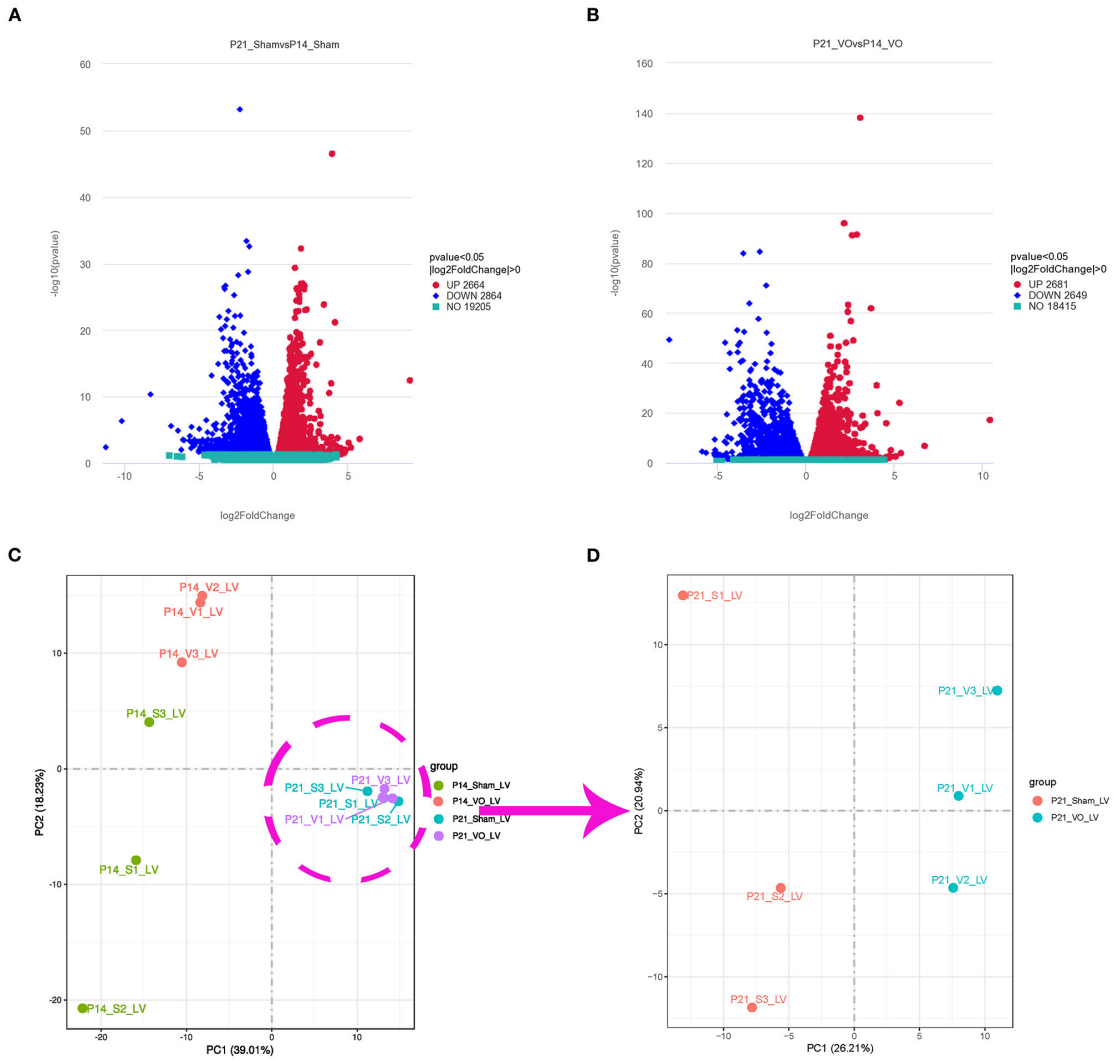
Transcriptomic changes in postnatal left ventricular (LV) development. **(A)** Volcano map of differentially expressed genes (DEGs) of postnatal LV development in the normal condition (P21_Sham vs. P14_Sham). **(B)** Volcano map of DEGs of postnatal LV development under the influence of volume overload (VO) (P21_VO and P14_VO). **(C)** The principal component analysis (PCA) of DEGs of the VO and Sham groups at P14 and P21. **(D)** The PCA of DEGs of the VO and Sham groups at P21.

To investigate how VO alters gene expressions in postnatal LV development from P7 to P21, we selected the LVs of the hearts from ACF and sham-operated mice at P14 and P21 and performed RNA-seq. Our results showed that, during normal postnatal LV development, there were 5,528 differentially expressed genes (DEGs) between P21_Sham and P14_Sham, among which 2,664 were upregulated and 2,864 were downregulated (Figure 3A), respectively. Under the influence of VO, there were 5,330 noted DEGs between P21_VO and P14_VO, among which 2,681 were upregulated and 2,649 were downregulated (Figure 3B). The principal component analysis (PCA) showed that VO caused greater changes in gene expression profiles at P14 than at P21 (Figure 3C). Although VO led to fewer changes in the transcriptome at P21 than at P14, the individual mice in the VO group were differed noticeably from the mice in the Sham group at P21 (Figure 3D). These results indicate that LVs are more sensitive to VO at P14 than at P21, and, at P21, the VO-influenced LVs are still quite different from normal LVs in terms of gene expression.

### Common Developmental Processes Between Normal and VO-Influenced LVs at the Prepubertal Stage

**Figure 4:**
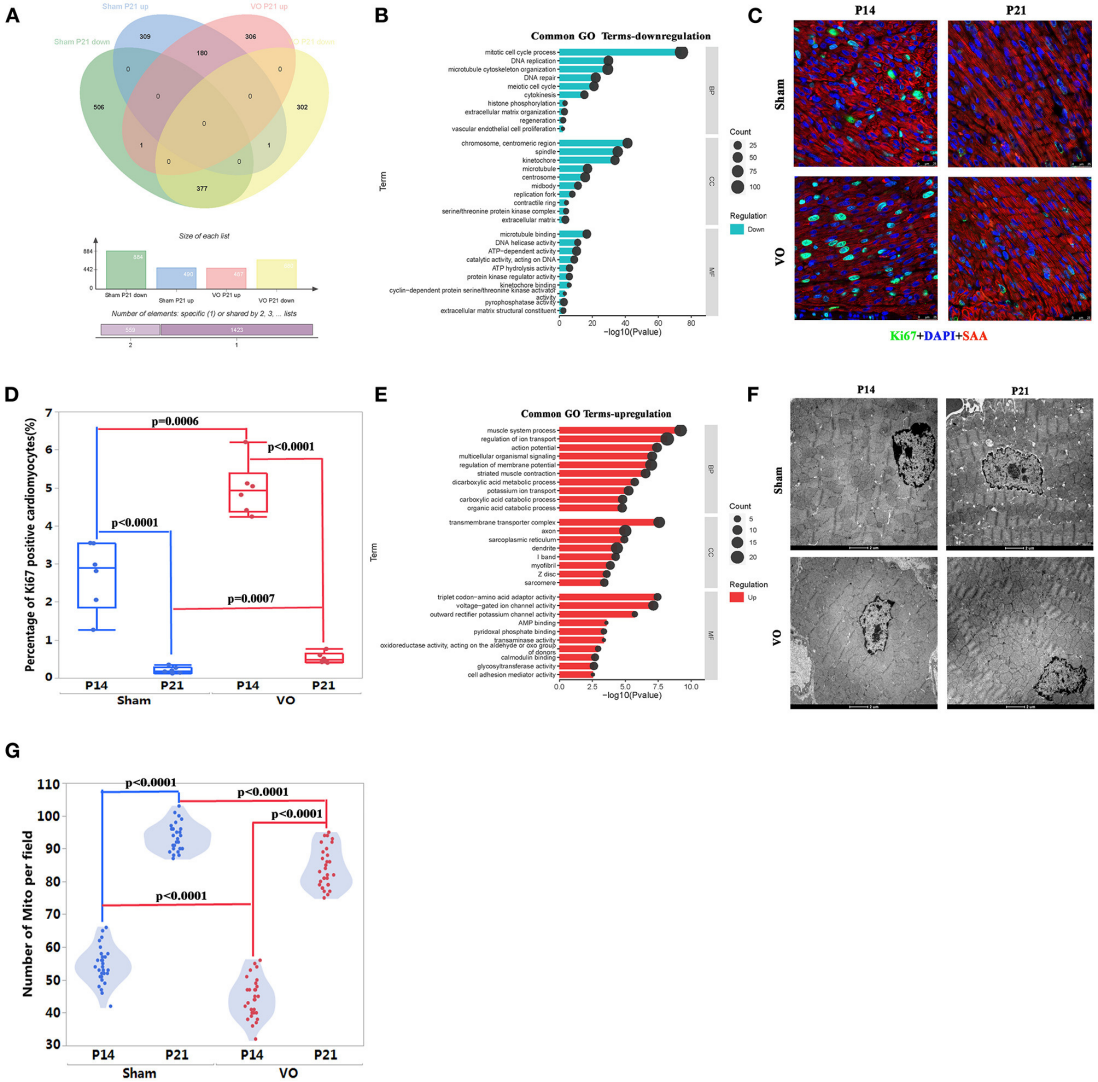
The common developmental processes between normal and volume overload (VO)-influenced left ventricles (LV). **(A)** The Venn analysis of differentially expressed genes (DEGs) in the two comparisons. There were 377 downregulated and 180 upregulated genes in common between the Sham comparison (P21_Sham vs. P14_Sham) and VO comparison (P21_VO vs. P14_VO), where 309 upregulated genes and 506 downregulated genes only appeared in the Sham comparison, and 306 upregulated genes and 302 downregulated genes only appeared in the VO comparison, respectively. **(B)** The common 377 downregulated genes were subjected to Gene Ontology (GO) enrichment analysis. The top 10 terms are displayed. The abscissa is the GO term, and the ordinate is the significance level of GO term enrichment. The higher the value, the more significant the result. The size of the dots represents the number of genes annotated to the GO term. BP, biological process; CC, cellular component; MF, molecular function. **(C)** Representative Ki67-positive cells. Ki67 (green); DAPI (blue); sarcomeric α-actinin (SAA, red). **(D)** Quantification of Ki67-positive cardiomyocytes. **(E)** The common 180 upregulated genes were subjected to GO enrichment analysis. **(F)** Representative transmission electron microscopy image of the LV. **(G)** Quantification of the number of mitochondria (Mito) from 30 fields of six mice in each group.

To further understand the common and different developmental processes between normal and VO-influenced LVs, we performed Venn analysis. The results showed that there were 377 downregulated and 180 upregulated genes in common between the Sham comparison (P21_Sham vs. P14_Sham) and VO comparison (P21_VO vs. P14_VO)—that is, 309 upregulated genes and 506 downregulated genes appeared only in the Sham comparison, and while 306 upregulated genes and 302 downregulated genes appeared only in the VO comparison (Figure 4A).

When these common downregulated genes were subjected to GO and KEGG pathway enrichment analysis, they were mainly associated with cell cycle regulation (Figure 4B, Supplementary Figure 2A). Cell cycle marker-Ki67 examination confirmed the results of GO and KEGG pathway enrichment analysis, revealing significant downregulation of Ki67-positive CMs in both comparisons (Figures 4C,D). It should be noted that the percentage of Ki67-positive CMs was higher in the VO than in the Sham group, either at P14 or P21, suggesting that VO contributes to cell cycle activities in the LV. These results were similar to those found in the RV, but the degree of VO-induced cell cycle activities in the LV was less than in the RV (13, 18).

The enriched analysis of the common upregulated genes demonstrated that they were mainly associated with metabolic maturation and cardiac muscle development (Figure 4E, Supplementary Figure 2B). Although cardiac maturation continued under VO, there were fewer Mito in the VO group than in the Sham group, either at P14 or P21 (Figures 4F,G). In addition, the proximity of Mito to sarcomere seemed less in the VO group than in the Sham group. These results suggest that VO affects the LV maturation, similar to as seen in the RV (5, 7).

### Unique Developmental Processes in Normal or VO-Influenced LVs at the Prepubertal Stage

**Figure 5:**
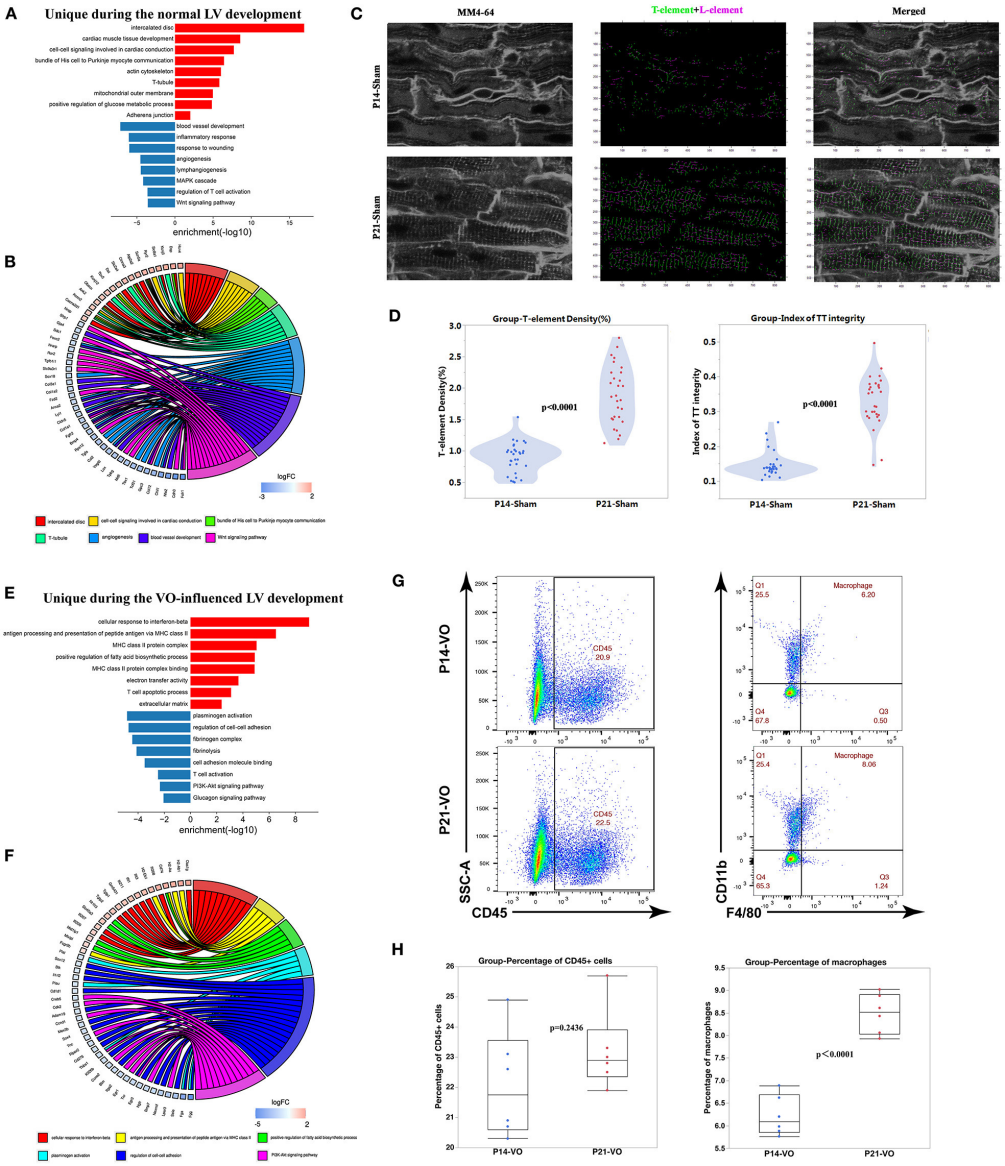
The unique developmental processes separating normal left ventricles (LVs) from volume overload (VO)-influenced LVs. **(A)** Genes uniquely appearing in the Sham comparison were subjected to GO and KEGG pathway analysis. **(B)** Circle plot of select genes indicated ontologies of Sham comparison. Gene expression relative difference (log2 fold change). **(C)** Left panel: representative of T-tubules from P14_Sham and P21_Sham groups. MM4-64 (T-tubule, white); middle panel: skeletalization of T-tubule system, T-element (green), and L-element (purple); right panel: merged. **(D)** Left panel: quantification of T-element density from 30 cardiomyocytes in each group; right panel: quantification of the index of TT integrity from 30 cardiomyocytes in each group. **(E)** Genes uniquely appearing in the VO comparison were subjected to GO and KEGG pathway analysis. **(F)** Circle plot of select genes indicated ontologies of VO comparison. Gene expression relative difference (log2 fold change). **(G)** Representative flow cytometry plots of CD45^+^ cells among singlets and macrophages (CD11b^+^F4/80^+^) among CD45^+^ cells. **(H)** Frequency quantification of the percentages of CD45+ cells and macrophages, *n* = 6, Student's *t*-test.

When the unique DEGs (309 upregulated and 506 downregulated genes), shown in Figure 4A of normal prepubertal LV development were subjected to GO and KEGG pathway enrichment analysis, the upregulated processes were mainly associated with cardiac conduction, such as the enriched terms of intercalated disc and T-tubule (Figures 5A,B), and the downregulated processes were mainly associated with angiogenesis and immune response (Figures 5A,B). The *in situ* T-tubule imaging confirmed an increase in T-element density and the index of TT integrity during normal LV development (Figures 5C,D).

The unique DEGs (306 upregulated genes and 302 downregulated genes, as shown in Figure 4A) of VO-induced prepubertal LV development were enriched in immune responses (Figures 5E,F), which were confirmed by the increase in macrophage percentages (Figures 5G,H).

These results suggested that VO may result in conduction disorders, which may be associated with the increase of immune responses. These were consistent with recent observations in human beings, showing that conduction disorders were associated with atrial VO, which induces sudden death in patients with AR (31, 32).

### Differences Between Normal and VO-Influenced LVs at P21

Since the postnatal developmental track of LVs was altered by VO, we compared P21_VO LVs with P21_Sham LVs to understand how the LVs were changed by VO at the end point of observation.

**Figure 6:**
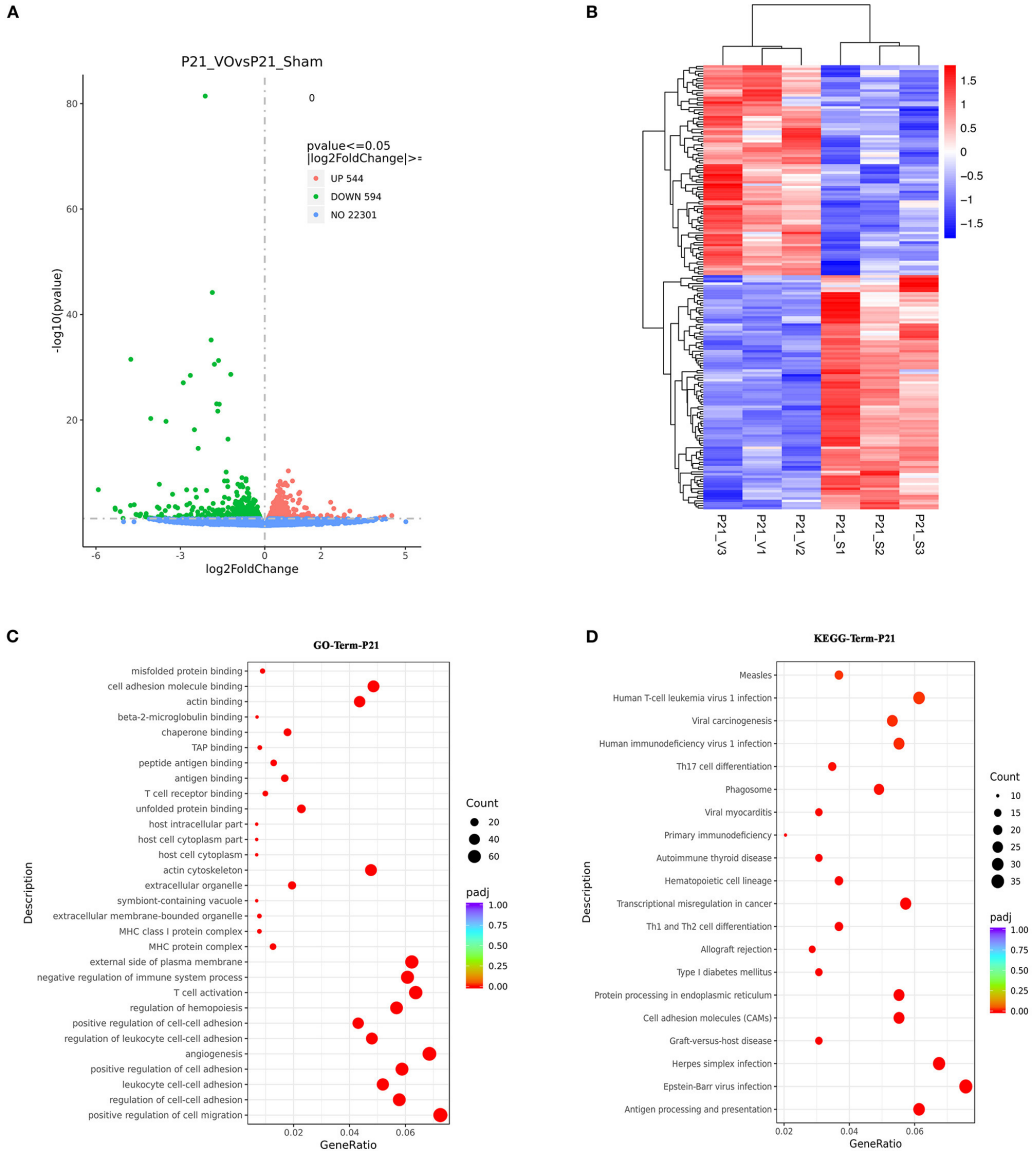
The molecular differences between normal and volume overload (VO)-influenced left ventricles (LVs) at P21. **(A)** Volcano map of differentially expressed genes (DEGs) between the normal and VO-influenced LVs at P21. **(B)** Cluster analysis of the DEGs between the normal and VO-influenced LVs. The clusters of genes in each group were quite different from each other but were similar within the same group. **(C)** Scatter plots of the enriched GO terms. From the results of the GO-enrichment analysis, we selected the 30 most significant terms to construct scatterplots for display. The size of the dots represents the number of genes annotated to the GO term, and the colors from red to purple represent the significance level of the GO term enrichment. **(D)** Scatterplot of the 20 most significant KEGG pathways. The abscissa is the ratio of the number of genes in the KEGG pathway analysis to the total number of differentially expressed genes, the ordinate is the KEGG pathway, the size of the dots represents the number of genes annotated to the KEGG pathway, and the colors from red to purple represent the significance level of KEGG pathway enrichment.

The results showed that there were 1,138 DEGs between the two groups, of which 594 were downregulated and 544 were upregulated (Figure 6A). When these genes were clustered, a heat map showed that the individual mice in the same group were similar to one another, yet differed markedly from those of the other group (Figure 6B). GO enrichment analysis of these DEGs demonstrated that the top 30 enriched terms were mainly associated with immune responses and angiogenesis (Figure 6C, Supplementary Figure 3A). KEGG pathway enrichment analysis of these DEGs demonstrated that the top 20 enriched terms were mainly associated with immune responses (Figure 6D, Supplementary Figure 3B). These results indicated that immune responses were activated in the LVs at P21 under the influence of VO.

### Verification of RNA-seq Results of P21 by Examination of Endothelial Intensity and Sarcomere Maturation

**Figure 7:**
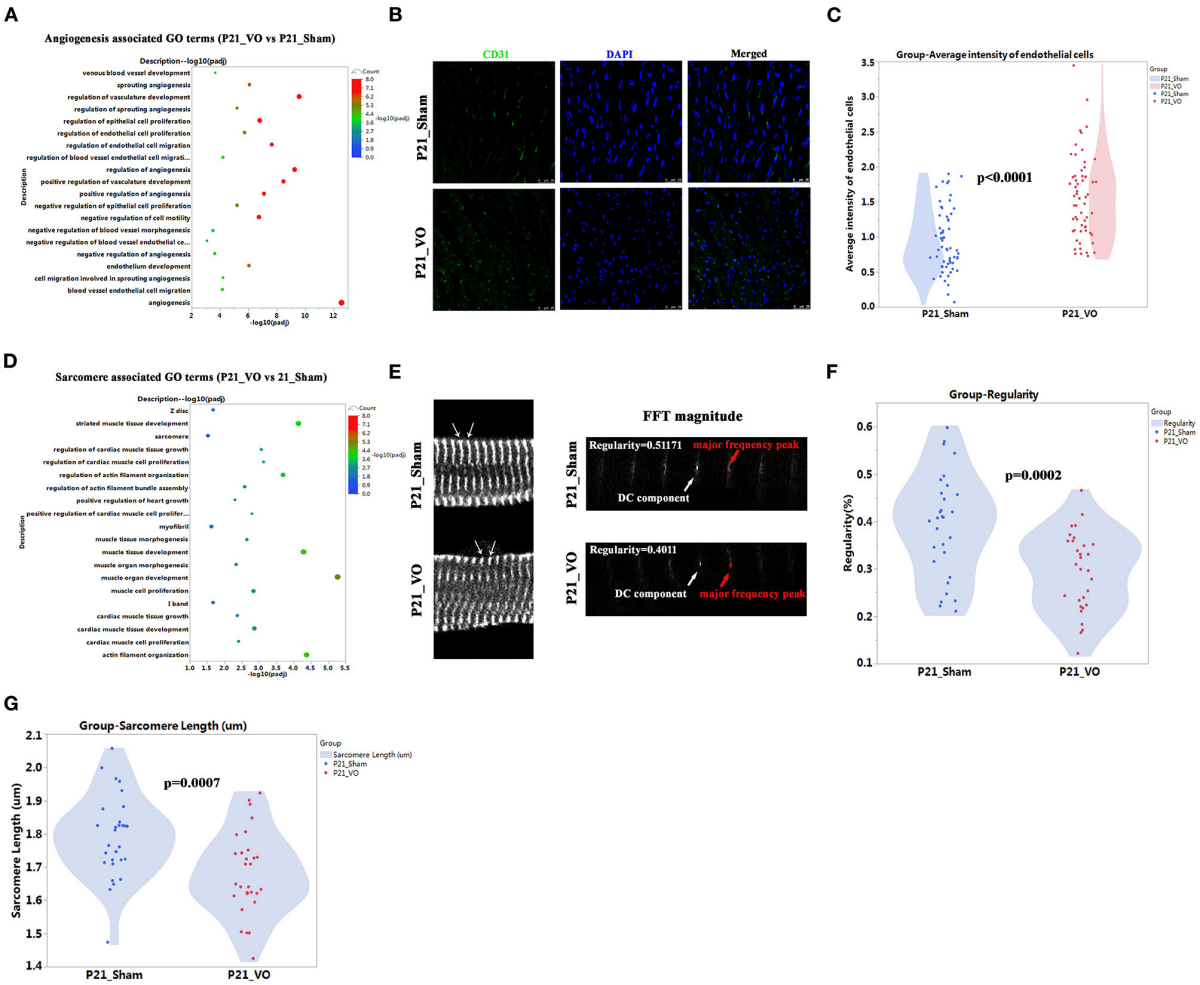
The different angiogenesis and sarcomere maturation between normal and volume overload (VO)-influenced left ventricles (LVs) at P21. **(A)** The angiogenesis associated GO terms from GO analysis of DEGs between normal and VO-influenced LVs at P21. **(B)** Representative CD31-positive cells; CD31 (green), DAPI (blue). **(C)** Quantification of CD31-positive cells, *n* = 60 sections from 6 mice in each group, Student's *t*-test. **(D)** The sarcomere associated GO terms from GO analysis of DEGs between normal and VO-influenced LVs at P21. **(E)** Left panel: representative of sarcomere organization from normal and VO-influenced LV at P21. Sarcomeric α-actinin (SAA, white), arrow indicated one sarcomere. Right panel: representative magnitude of fast Fourier transformation (FFT) of cardiomyocytes present at left panel; arrows indicated the major frequency peak, and the direct current (DC) component was defined as the transformed series at frequency 0, which represents the summation of signals of all pixels in the image; The major frequency was defined as the second highest peak; regularity is defined as the magnitude of the major frequency normalized to that of DC component. **(F)** Quantification of the sarcomere regularity from *n* = 30 cardiomyocytes in each group, Student's *t*-test. **(G)** Quantification of the sarcomere length from *n* = 30 cardiomyocytes in each group, Student's *t*-test.

Our previous publications indicate that angiogenesis is one of the major RV responses to VO at the prepubertal stage (13, 18) and the major enriched GO terms at P21 (Figure 6C, Supplementary Figure 3A). Thus we checked the enriched terms associated with angiogenesis. As shown in Figure 7A, there were 20 significantly enriched GO terms associated with angiogenesis. The average intensity of endothelial cells significantly increased in P21_VO when compared to in P21_Sham (Figures 7B,C).

Our previous publications also indicated that RV maturation is affected by VO (13, 20) and the maturation markers, Mito and T-tubule, in LV are affected by VO (Figures 4F,G, 5D). To further confirm that LV maturation was affected by VO, we checked another maturation marker, sarcomere, at P21. The results showed that there were couples of significant enriched GO terms associated with sarcomere (Figure 7D). The sarcomere regularity and length were reduced in P21_VO when compared to in P21_Sham (Figures 7E–G, Supplementary Table S2). One of the major hallmarks of cardiomyocyte maturation is myofibril maturation, which is characterized by improved sarcomere alignment and increased sarcomere length (19). On postnatal day 21, normal myofibrils were mature with regular and proper long sarcomeres, while VO group myofibrils stayed immature with shorter and less regular sarcomeres. These results indicate that LV maturation is also affected by VO.

## Discussion

Pediatric AR is a common entity with both preoperative and postoperative congenital heart disease, such as previous intervention for congenital aortic valve stenosis or primary disease of the congenital bicuspid valve and the aortic root or ascending aorta (33–35). AR generally produces VO, and our previous studies have demonstrated that VO affects RV maturation (12, 13, 18). Whether and how VO manipulates LV maturation remains unexplored. In addition, current guidelines to guide intervention timing for asymptomatic AR were developed for adult patients, which is now under controversy (4, 12, 33). Available information about intervention timing for asymptomatic pediatric AR is limited (33, 36). The current study firstly demonstrated that VO affects LV maturation, suggesting the need for early intervention for pediatric AR when considering LV maturation as an index. The results showed that even mild-to-moderate AR in pediatric patients with no heart failure sign can significantly influence the heart development and postpone cardiomyocyte maturation. Although the best time for aortic valve intervention in pediatric chronic AR is currently controversial, the main source of concern is the postoperative complications and management strategies needed for valve repair or replacement. The current study suggests that the LV VO condition should be corrected as soon as possible to prevent an impact on heart development. The intervention method is being developed, and it is hoped that researchers will 1 day find a way to correct the AR with few complications and no need for coagulation.

An important question awaiting answers is how VO deters LV maturation. The transcriptome results showed that VO led to changes in genes associated with sarcomere, Mito, T tubules, and intercalated disc. The gene changes affected normal LV function by several following contributions. First, altered expression of critical sarcomere isoforms modified the contractile properties of CMs (37). Mito increase in number and size and became well organized with respect to sarcomeres, determining the switch of energy metabolism in CMs from glycolysis to oxidative phosphorylation, which provides sufficient energy for the contraction of CMs and the conduction of electrical signals (38). The establishment of T-tubules and intercalated disc permitted rapid action potential penetration with enlarged CMs, which was required for proper conduction of electrical signals (39). As shown in Figure 6D and Supplementary Figure 3B, the top 20 enriched terms of KEGG pathway analysis were associated with immune responses. Recent studies have demonstrated that immune responses underlie the mechanisms of cardiac regeneration and repair (40, 41), and VO also initiates an immune response in the neonatal RV (26). Immune cells, such as macrophages, are required for Mito homeostasis. Anomalous Mito in cardiomyocytes lead to metabolic alterations and ventricular dysfunction (42). It has been reported that metabolic maturation is a key driver of sarcomeric and electrophysiology maturation (20, 43). Thus, it is possible that the immune response modulates cardiomyocyte structure and function by regulating Mito function. In all, the above results suggest that immune responses may play a critical role in the regulation of LV maturation under the influence of VO.

Another important concern of the current study is the hemodynamic differences between ACF-induced and AR-induced VO. Do prepubertal LVs respond to these two kinds of VO similarly, and to what extent can ACF-induced VO simulate AR-induced VO? Because there is limited space for surgical creation of AR-induced VO in prepubertal mice or rats, large animals such as swine may be adopted to answer the above questions. Additionally, in adult animals, hemodynamic responses and remodeling in RV between ACF-induced and AR-induced VO were quite similar (44), and multiple studies have used ACF modeling to explore the molecular mechanisms of LV in response to VO (24, 25), providing insights to understand how LV responds to VO. Thus, the current prepubertal VO mouse model may be a not bad choice for understanding how VO affects postnatal LV development.

Another important finding is that VO has a smaller effect on the LV than on the RV. Our results showed that, at P14, under the influence of VO, the fold change of Ki67-positive CMs was 1.67 in the LV (Figures 4C,D), yet the same is 19.7 in the RV (18). Correspondingly, under the influence of VO, at P21, the fold change of sarcomere regularity was 0.74 in the LV (Figures 7E,F), but has been reported to be 0.56 in the RV (13). These results suggest that the RV was more sensitive to VO than the LV in terms of the cell cycle and maturation. A true AR model would be preferable to evaluate how VO impacts the LV and RV, but it is impossible to create with P7 mice using current equipment. We hope that, in further research, aortic valve puncture-induced VO will be created in neonatal large animals to compare with an ACF-VO model to understand the differences between these models. The RV is also more sensitive to pressure overload than the LV in terms of the cell cycle at neonatal stage (45, 46).

In summary, the current study first demonstrated that VO poses an effect on LV maturation, suggesting the crucial need for an early correction of pediatric AR, and the underlying mechanisms may be associated with immune responses. Separately the differences between the effects of ACF-induced and AR-induced VO on prepubertal LVs must be analyzed in large animals.

## Data Availability Statement

The datasets presented in this study can be found in online repositories. The names of the repository/repositories and accession number(s) can be found in the article/Supplementary Material.

## Ethics Statement

The animal study was reviewed and approved by Animal Welfare and Human Studies Committee at Shanghai Children's Medical Center.

## Author Contributions

YH, SS, LY, and FL designed the study. YH, SS, CZ, and DL performed the experiments. YH, CJ, YX, and LC collected the samples. YH, FL, and LY conducted the statistical analysis. YH, LY, HZ, and FL wrote the manuscript. FL, JL, HH, and LY reviewed and edited the manuscript. All authors read and approved the final manuscript.

## Funding

This work was supported by the Shanghai Science and Technology Innovation Project (No. 19411950200), the Clinical Science and Technology Innovation Project of Shanghai Shenkang Hospital Development Center (No. SHDC12018128), the Guided Project Fund of Shanghai Science and Technology Commission (No. 20Y11910400), the Key Discipline Group Development Fund of Health and Family Planning Commission of Pudong New District (No. PWZxq2017-14), the National Natural Science Foundation of China (Nos. 82001835 and 81800285), the National Key R&D Program of China (No. 2019YFA0110401), the Science and Technology Innovation Action Plan of Shanghai—Experimental Animal Research (No. 201409005900), the Foundation of Pudong Science and Technology Development (No. PKJ2019-Y12), and the Program for Outstanding Medical Academic Leader in Shanghai (HZ).

## Conflict of Interest

The authors declare that the research was conducted in the absence of any commercial or financial relationships that could be construed as a potential conflict of interest.

## Publisher's Note

All claims expressed in this article are solely those of the authors and do not necessarily represent those of their affiliated organizations, or those of the publisher, the editors and the reviewers. Any product that may be evaluated in this article, or claim that may be made by its manufacturer, is not guaranteed or endorsed by the publisher.

## Abbreviations

VO: Volume overload
GO: Gene Ontology
KEGG: Kyoto Encyclopedia of Genes and Genomes
AR: Aortic regurgitation
RNA-seq: RNA sequencing
ACF: Aortocaval fistula
H&E: Hematoxylin and eosin
FPKM: Fragments Per Kilobase of transcript sequence per Million
PBS: Phosphate buffer saline
DAPI: 4′, 6-diamidino-2-phenylindole
LV: Left ventricle
CM: Cardiomyocyte
CHD: Congenital heart disease
Mito: Mitochondrion
AoV: Aortic valve
AA: Abdominal aorta
IVC: Inferior vena cava
VTI: Velocity time integral
PCR: Polymerase chain reaction
DEG: Differentially expressed gene.
