# Supplementary material for: Molecular Changes in Prepubertal Left Ventricular Development Under Experimental Volume Overload
Source: Front Cardiovasc Med. 2022 Apr 12;9:850248. doi: 10.3389/fcvm.2022.850248 (PMC9039316; doi:10.3389/fcvm.2022.850248)
Supplement: Supplementary file 2 [file Table_2.DOCX]

Supplemental Table S2. Values for the sarcomere regularity and length from n=30 cardiomyocytes in each group

| Number | Sarcomere Length (um) | Regularity | Group |
| --- | --- | --- | --- |
| 1 | 1.8114 | 0.4103 | P21_Sham |
| 2 | 1.765 | 0.54393 | P21_Sham |
| 3 | 1.8356 | 0.23009 | P21_Sham |
| 4 | 1.8227 | 0.40879 | P21_Sham |
| 5 | 1.7094 | 0.23249 | P21_Sham |
| 6 | 1.93 | 0.45654 | P21_Sham |
| 7 | 1.825 | 0.44685 | P21_Sham |
| 8 | 1.8198 | 0.28216 | P21_Sham |
| 9 | 1.7464 | 0.24722 | P21_Sham |
| 10 | 1.6325 | 0.38481 | P21_Sham |
| 11 | 1.7424 | 0.42 | P21_Sham |
| 12 | 1.7608 | 0.4233 | P21_Sham |
| 13 | 1.825 | 0.40747 | P21_Sham |
| 14 | 1.824 | 0.5631 | P21_Sham |
| 15 | 1.659 | 0.4957 | P21_Sham |
| 16 | 1.663 | 0.4594 | P21_Sham |
| 17 | 1.999 | 0.3658 | P21_Sham |
| 18 | 1.474 | 0.5978 | P21_Sham |
| 19 | 1.7137 | 0.5687 | P21_Sham |
| 20 | 1.875 | 0.3457 | P21_Sham |
| 21 | 1.958 | 0.2216 | P21_Sham |
| 22 | 1.6486 | 0.4758 | P21_Sham |
| 23 | 1.7216 | 0.4197 | P21_Sham |
| 24 | 1.9661 | 0.3514 | P21_Sham |
| 25 | 1.882 | 0.3155 | P21_Sham |
| 26 | 2.057 | 0.48788 | P21_Sham |
| 27 | 1.8255 | 0.21079 | P21_Sham |
| 28 | 1.8233 | 0.2704 | P21_Sham |
| 29 | 1.72398 | 0.3347 | P21_Sham |
| 30 | 1.7215 | 0.4009 | P21_Sham |
| 31 | 1.649 | 0.3384 | P21_VO |
| 32 | 1.6243 | 0.21723 | P21_VO |
| 33 | 1.5945 | 0.3487 | P21_VO |
| 34 | 1.751 | 0.16579 | P21_VO |
| 35 | 1.727 | 0.29626 | P21_VO |
| 36 | 1.633 | 0.4653 | P21_VO |
| 37 | 1.8062 | 0.3307 | P21_VO |
| 38 | 1.7426 | 0.2204 | P21_VO |
| 39 | 1.7243 | 0.2337 | P21_VO |
| 40 | 1.6229 | 0.2535 | P21_VO |
| 41 | 1.62 | 0.372 | P21_VO |
| 42 | 1.505 | 0.4145 | P21_VO |
| 43 | 1.425 | 0.1708 | P21_VO |
| 44 | 1.8477 | 0.39059 | P21_VO |
| 45 | 1.901 | 0.3914 | P21_VO |
| 46 | 1.572 | 0.3586 | P21_VO |
| 47 | 1.709 | 0.3238 | P21_VO |
| 48 | 1.923 | 0.1831 | P21_VO |
| 49 | 1.6403 | 0.2991 | P21_VO |
| 50 | 1.6403 | 0.2238 | P21_VO |
| 51 | 1.7974 | 0.3309 | P21_VO |
| 52 | 1.502 | 0.3589 | P21_VO |
| 53 | 1.5017 | 0.24337 | P21_VO |
| 54 | 1.7403 | 0.2395 | P21_VO |
| 55 | 1.7088 | 0.3515 | P21_VO |
| 56 | 1.6135 | 0.2109 | P21_VO |
| 57 | 1.889 | 0.3655 | P21_VO |
| 58 | 1.6223 | 0.2785 | P21_VO |
| 59 | 1.7297 | 0.3096 | P21_VO |
| 60 | 1.621 | 0.1217 | P21_VO |
