# Supplementary figures and images for: Molecular Changes in Prepubertal Left Ventricular Development Under Experimental Volume Overload
Source: Front Cardiovasc Med. 2022 Apr 12;9:850248. doi: 10.3389/fcvm.2022.850248 (PMC9039316; doi:10.3389/fcvm.2022.850248)

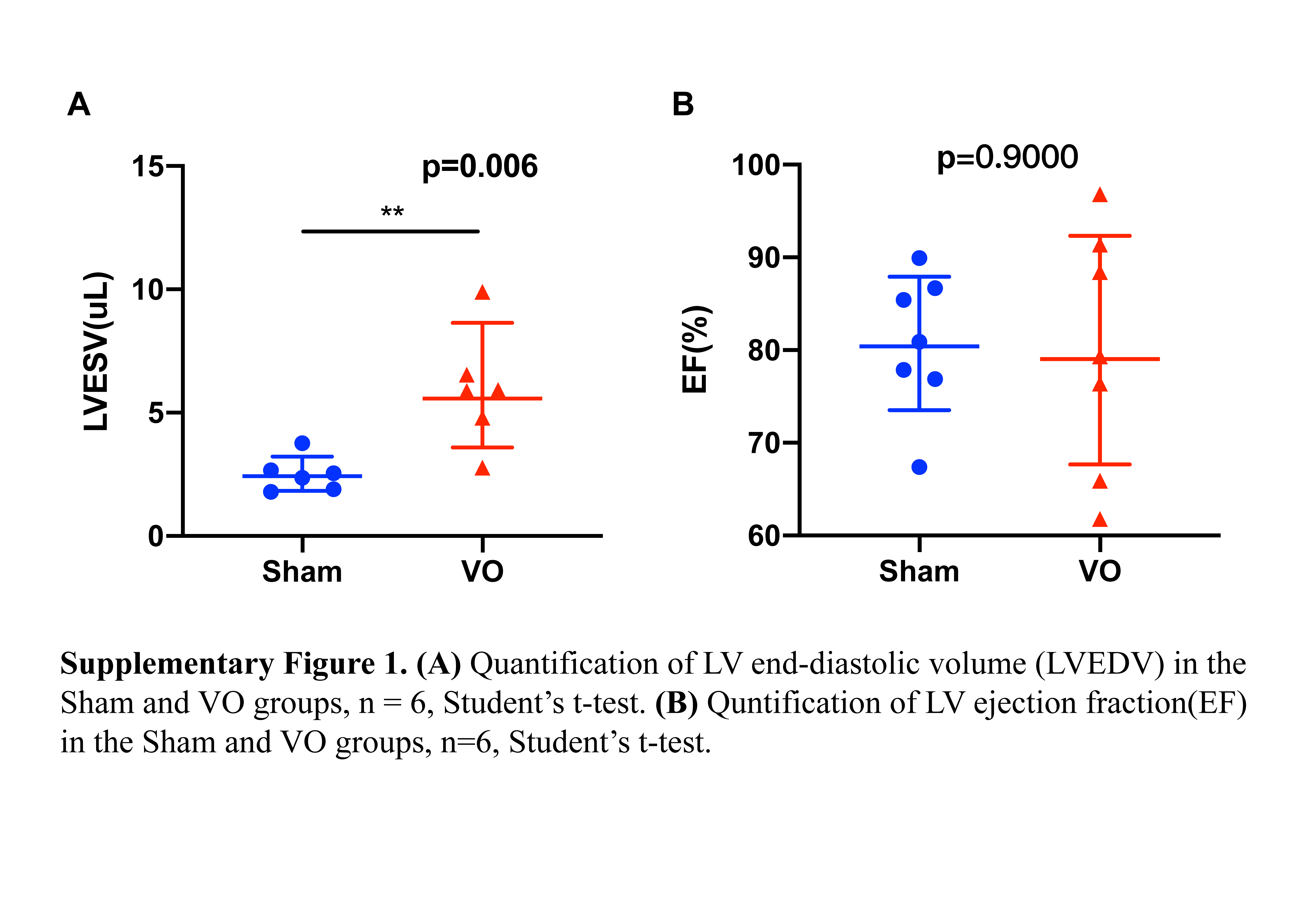

Supplement: Supplementary file 3 [file Image_1.TIF]

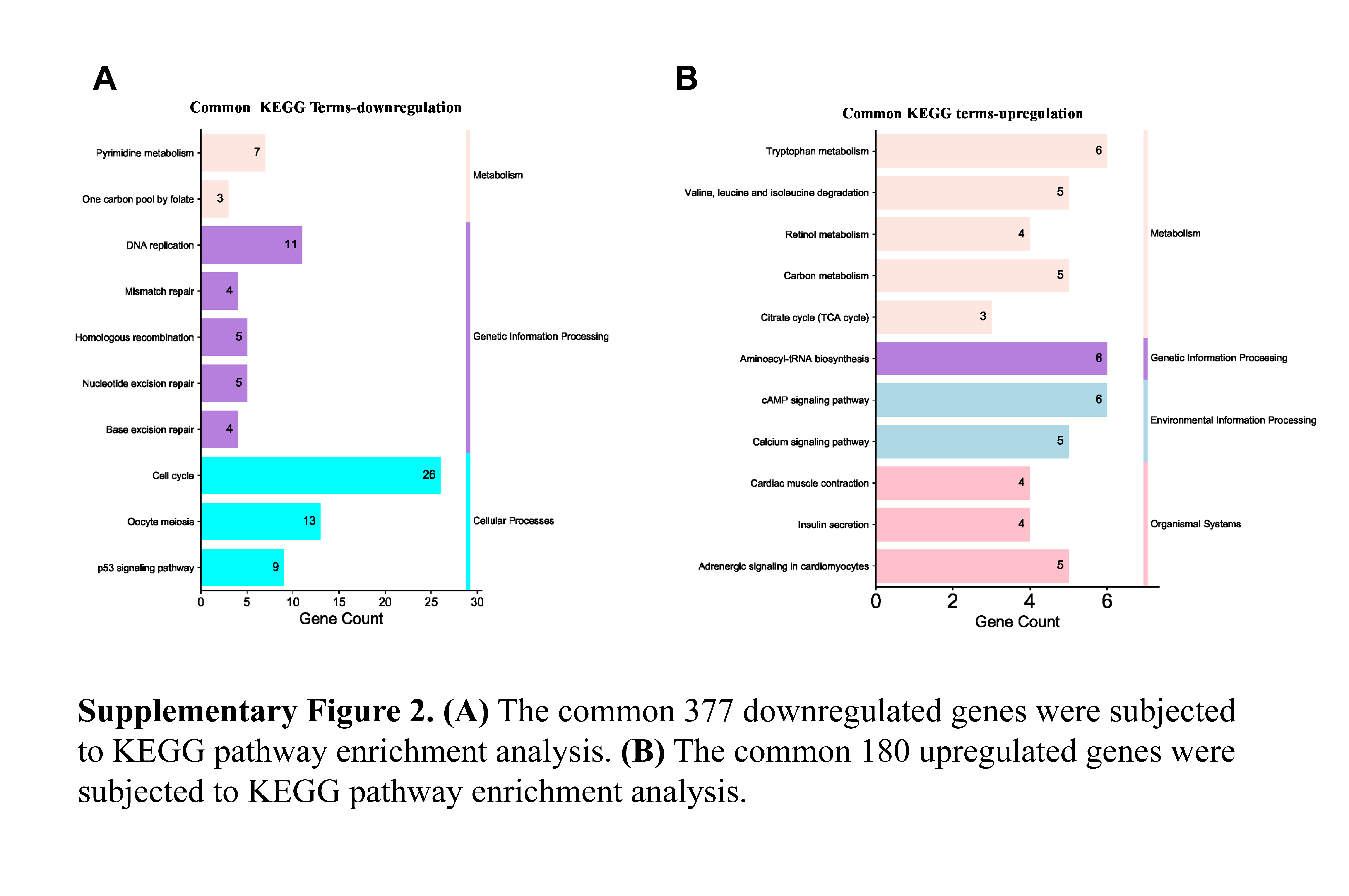

Supplement: Supplementary file 4 [file Image_2.TIF]

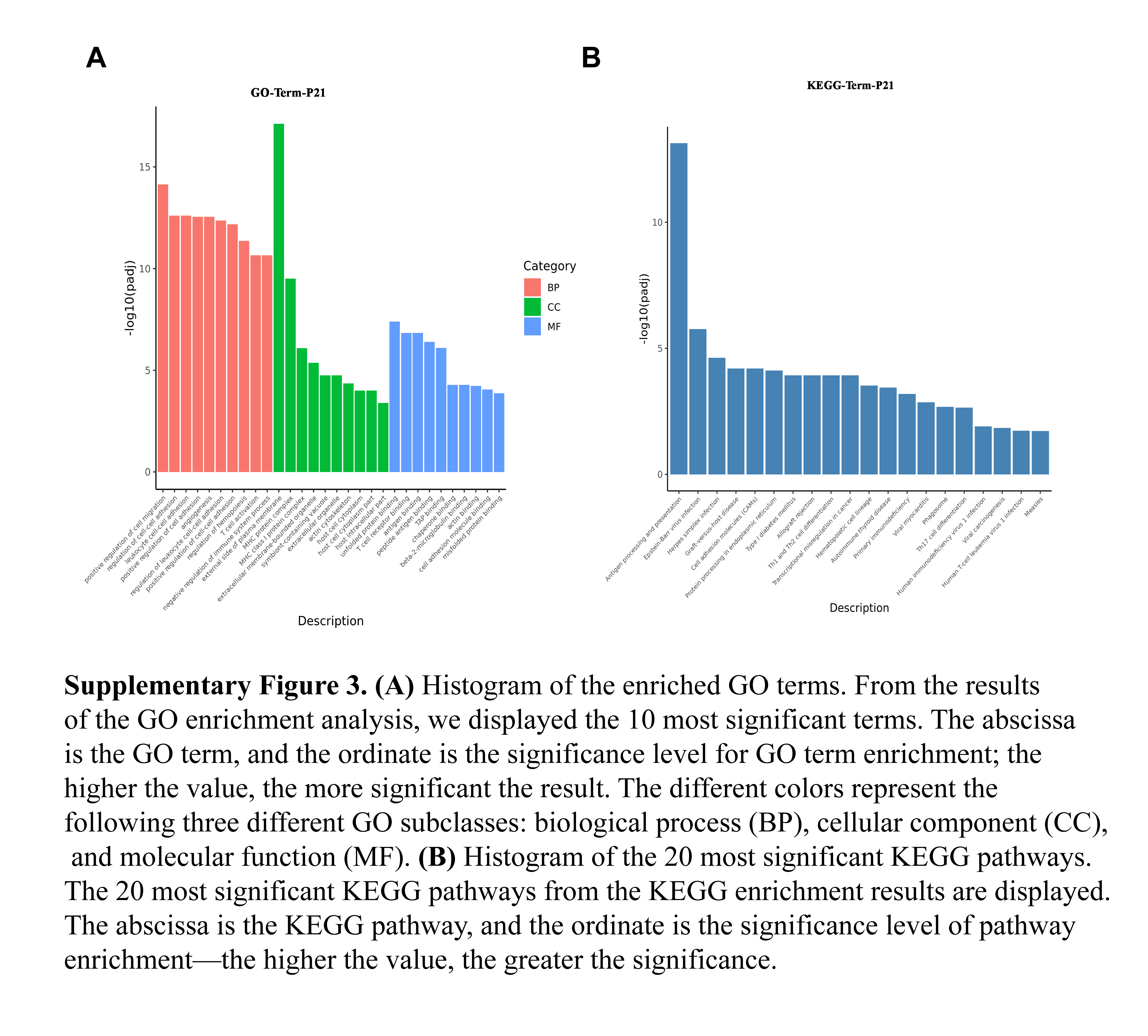

Supplement: Supplementary file 5 [file Image_3.TIF]
